# Therapeutic Cancer Vaccines: Mechanisms, Clinical Progress, and Future Directions

**DOI:** 10.3390/vaccines14070599

**Published:** 2026-07-07

**Authors:** Kendra Wilson, Jesus Salvador Flores Banda, Sanjana Bukkapatnam, Fatima Raza, Erminia Massarelli

**Affiliations:** 1 Department of Internal Medicine, Division of Hematology and Medical Oncology, University of Texas at Tyler School of Medicine, 11937 US-271, Tyler, TX 75708, USA; kendra.wilson@uttyler.edu (K.W.); fatima.raza@uttyler.edu (F.R.); 2 Department of Internal Medicine, Division of Hematology and Oncology, SUNY Downstate Health Sciences University, 445 Lenox Rd., Brooklyn, NY 11203, USA; jesus.floresbanda@downstate.edu; 3 Department of Internal Medicine, Roswell Park Comprehensive Cancer Center, 665 Elm St., Buffalo, NY 14203, USA; sanjana.bukkapatnam@roswellpark.com

**Keywords:** therapeutic cancer vaccines, autologous tumor cell vaccines, allogeneic tumor cell vaccines, dendritic cell vaccines, peptide-based vaccines, nucleic acid vaccines, viral vector-based vaccines, bacterial-based vaccines

## Abstract

Therapeutic cancer vaccines have emerged as a promising approach in cancer immunotherapy, aiming to stimulate the immune system to recognize and eliminate malignant cells. Despite this potential, clinical efficacy has remained variable across multiple vaccine platforms. This review synthesizes completed clinical trials evaluating major therapeutic cancer vaccine modalities, including peptide-based, nucleic acid--based, dendritic cell, whole cell, and viral and bacterial vector--based vaccines, with a focus on safety, immunogenicity, and clinical outcomes for diverse tumor types. Overall, these vaccines demonstrate a favorable safety profile; however, clinical efficacy as monotherapy has been limited. Clinical outcomes vary by platform and tumor type and are influenced by factors such as antigen selection, tumor heterogeneity and immunosuppressive tumor microenvironment (TME). Recent advances in antigen design, vaccine technologies, and combination strategies are redefining the role of therapeutic cancer vaccines in oncology. Future progress will depend on optimizing their integration with standard treatment modalities, particularly immune checkpoint inhibitors.

## 1. Introduction

According to the American Cancer Society, an estimated 2,041,910 new cancer cases are expected for 2025, along with 618,120 cancer deaths [1]. The estimated national cost for cancer care in the United States is projected to increase by 34% from 2015 to 2030, reaching an estimated cost of $246 billion [2]. Despite major advances in surgery, radiation, targeted therapies, and immune checkpoint inhibitors, many patients with advanced malignancies still experience relapse or progression, underscoring the need for more effective, durable, and cost-efficient treatment strategies. Therapeutic cancer vaccines have emerged as a new therapeutic approach, designed to mobilize the host immune system against tumor cells.

Cancer vaccines are designed to educate the immune system to recognize and eliminate tumor cells. Effective antitumor immunity relies on the coordinated activity of both the innate and adaptive immune systems. The innate immune response serves as the first line of defense against malignant transformation and consists of components such as dendritic cells (DCs), macrophages, natural killer cells, and pattern recognition receptors. This arm of immunity is activated by tumor-associated antigens (TAAs) or danger-associated molecular patterns released by stressed or dying tumor cells [3]. In the context of cancer vaccination, innate immune activation most commonly occurs through antigen presenting cells (APCs) recognizing tumor antigens, leading to cytokine secretion and localized inflammation [3]. This early innate response is essential for the initiation and shaping of subsequent adaptive immunity [4,5].

Adaptive immunity provides durable, antigen-specific protection mediated by T and B lymphocytes and plays a central role in immunosurveillance, the process by which the immune system detects and eliminates emerging malignant cells [6]. Tumors, however, possess multiple mechanisms to evade immune recognition and destruction, thereby enabling disease progression. Elucidation of these immune evasion strategies has led to the development of immunotherapies such as immune checkpoint inhibitors. Despite their success, a significant proportion of patients either fail to respond or develop acquired resistance over time.

The rationale for cancer vaccines is grounded in their capacity to activate both innate and adaptive immune responses to recognize, target, and eliminate tumor cells. Vaccine-induced immune activation begins with the delivery of TAAs, which may be introduced through a variety of platforms including whole tumor cells, peptides, proteins, deoxyribonucleic acid (DNA), messenger ribonucleic acid (mRNA), antigen-loaded DCs, or tumor-derived genetic material. Following antigen delivery, APCs process tumor-derived peptides and present them on major histocompatibility complex (MHC) molecules, leading to the activation of CD4^+^ helper and CD8^+^ cytotoxic T cells [7].

Activated cytotoxic T lymphocytes directly mediate tumor cell killing, while CD4^+^ helper T cells produce cytokines that support cytotoxic T cell expansion and function. In addition, helper T cells facilitate B cell activation and antibody production, further contributing to antitumor immunity [8]. Together, the coordinated engagement of innate and adaptive immune mechanisms underpins the therapeutic potential of cancer vaccines. These immunologic principles have driven the development of diverse vaccine platforms, many of which are currently being evaluated in clinical trials. In this review, we summarize key completed and ongoing studies across the major classes of therapeutic cancer vaccines.

## 2. Vaccine Platforms

### 2.1. Cell-Based Vaccines

#### 2.1.1. Autologous Tumor Cell Vaccines

Autologous cancer vaccines are generated from a patient’s own tumor or immune cells, thereby offering a personalized therapy designed to harness the unique antigenic features of their malignancy [9]. By utilizing patient-specific tumor material, these individualized vaccines expose the immune system to a broad range of TAAs rather than focusing on a single predefined target, enabling subsequent immune responses to reflect both intratumoral and intertumoral heterogeneity, allowing for more effective and individualized therapies [10].

Clinical trials investigating autologous cancer vaccines in melanoma, renal cell carcinoma (RCC), and glioblastoma multiforme have consistently demonstrated induction of antitumor immune responses, although clinical efficacy has remained variable. In melanoma, heat-shock protein--based vaccines such as the Heat-Shock Protein-Peptide Complex 96 (HSPPC 96; Oncophage) were shown in early-phase studies to elicit tumor-specific immune responses with a favorable safety profile; however, their clinical benefit was limited [11]. Despite successful immunogenicity, the magnitude and functionality of cytotoxic T cell responses were insufficient to overcome the immunosuppressive TME. Consequently, immune activation did not consistently translate into objective tumor regression or durable clinical benefit [11].

Similar observations were reported in RCC. Early-phase trials of HSPPC 96 demonstrated the ability to generate tumor-specific immune responses with acceptable safety. However, in a randomized phase 3 adjuvant trial conducted after nephrectomy, vaccination with HSPPC 96 failed to achieve a statistically significant improvement in overall survival (OS) compared with observation alone [12].

In contrast, more favorable outcomes have been observed with certain autologous whole-cell vaccine approaches. In a randomized phase 3 trial evaluating an autologous tumor cell vaccine combined with Bacillus Calmette-Guérin (BCG) in resected stage II colon cancer (OncoVAX), stage-stratified analyses demonstrated a significant improvement in recurrence-free survival (RFS) at five years (21.3% vs. 37.7% events; *p* = 0.008), along with a corresponding improvement in OS (5-year mortality 17.5% vs. 27.3%; *p* = 0.014) [13]. Similarly, a randomized phase 2 trial of an autologous formalin fixed tumor vaccine in patients with resected hepatocellular carcinoma showed significant improvements in both RFS (*p* = 0.003) and OS (*p* = 0.01) compared with no adjuvant therapy [14].

**Figure 1:**
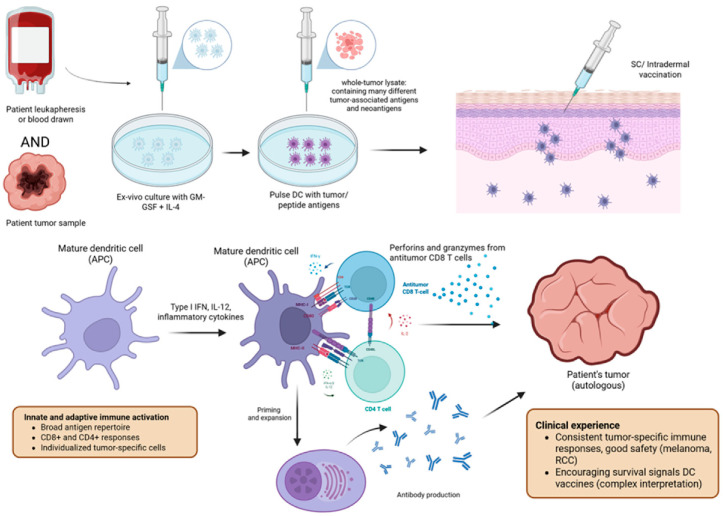
Mechanism of action for autologous tumor cell vaccine. Created in BioRender. Flores Banda, J. (2026). https://BioRender.com/pllux4u (accessed on 8 February 2026). GM-CSF: Granulocyte-macrophage colony-stimulating factor, IL-4: Interleukin-4, DC: dendritic cell, SC: subcutaneous, APC: Antigen-presenting cell, IFN: Interferon, IL-12: Interleukin-12.

Taken together, these findings indicate that autologous tumor-based vaccines can reliably induce tumor-specific immune responses across multiple malignancies and are generally associated with favorable safety profiles. However, their clinical utility has been limited by inconsistent therapeutic efficacy, a lack of durable overall clinical benefit, and the dominant immunosuppressive influence of the TME, particularly when these approaches are employed as monotherapy. Figure 1 below outlines the mechanism of autologous tumor cell vaccines.

#### 2.1.2. Allogeneic Tumor Cell Vaccines

Allogeneic tumor vaccines represent an alternative antigen source for therapeutic cancer vaccination, using tumor cells or derivatives from donors rather than from the individual patient. Initial development of allogeneic vaccines stemmed from melanoma research. In 1985, Liveingston et al. reported a serological response in melanoma patients receiving allogeneic melanoma cell vaccines [15]. Sixteen of seventeen patients were reported to have developed antibodies against alloantigens present on the vaccine cells.

**Figure 2:**
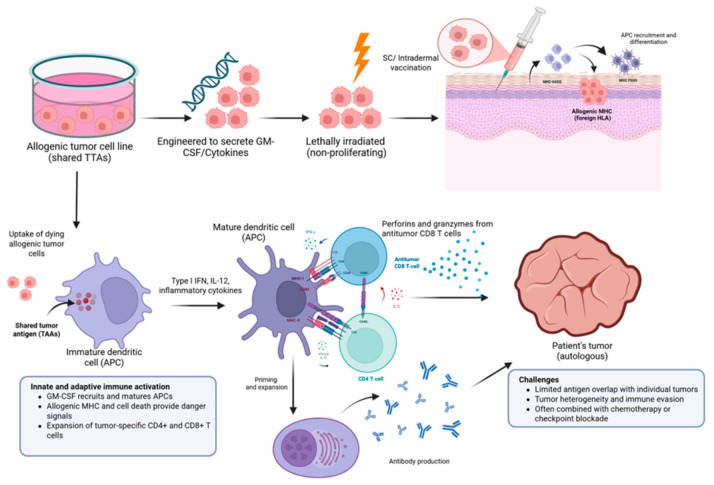
Mechanism of action for allogeneic vaccine. Created in BioRender. Flores Banda, J. (2026). https://BioRender.com/520esfc (accessed on 8 February 2026). TAAs: Tumor-associated antigens, GM-CSF, DC, SC, APC, IFN, IL-12.

Allogeneic cancer vaccines function by presenting TAAs from donor tumor cells to activate a patient’s immune response [16]. Figure 2 below outlines the mechanism of immune activation of an allogeneic vaccination. These vaccines offer advantages in standardization and ease of production. Although specific mechanisms vary by platform, allogeneic vaccines generally act by activating host APCs and priming tumor-specific T cells. Multiple allogeneic platforms have been developed, including whole tumor cell vaccines, allogeneic dendritic cell vaccines, and novel allogeneic platforms such as allogeneic chimeric antigen receptor-engineered cells.

Clinical trials investigating allogeneic vaccines for the treatment of breast cancer, chronic myeloid leukemia, lung cancer, melanoma, pancreatic cancer, prostate cancer, colon cancer and renal cancer have been published [16]. Here, we briefly summarize key findings from selected allogeneic tumor vaccine trials.

Giaccone et al. conducted a phase 3 study evaluating belagenpumatucel-L (an allogeneic tumor cell vaccine) as maintenance therapy for non-small cell lung cancer. There was no significant difference in survival, with median OS of 20.3 months versus 17.8 months (Hazard ratio (HR) 0.94, *p* = 0.594) in the belagenpumatucel-L and placebo groups, respectively [17]. Although a trend toward improved survival was observed in patients randomized within 12 weeks of chemotherapy or prior radiation. Faries et al. conducted a phase 3 study evaluating an allogeneic whole-cell vaccine in combination with BCG following complete resection of stage IV melanoma [18]. No improvements in outcomes were observed, with a median 5-year OS of 34.9 months versus 39.1 months (HR 1.053, *p* = 0.696) in the allogeneic vaccine and placebo groups, respectively. Wu et al. reported a phase 2 study of an allogeneic granulocyte-macrophage-colony-stimulating factor (GM-CSF)-transfected pancreatic tumor vaccine (GVAX) combined with ipilimumab as maintenance treatment for metastatic pancreatic cancer [19]. GVAX plus ipilimumab did not improve OS (median OS 9.38 months versus 14.7 months for GVAX versus standard of care; HR 1.75, *p* = 0.019). In contrast, Biavati et al. reported encouraging activity in a proof-of-principle clinical trial of an allogeneic multiple myeloma GM-CSF-secreting vaccine in combination with lenalidomide [20]. At a median follow-up of five years, median OS was 7.8 years from enrollment (95% confidence interval (CI): 4.2–7.8 years), and eight patients (53%) had a true complete response.

As illustrated by the trials summarized above, additional investigation is required to achieve meaningful clinical benefit with this class of vaccines, whether administered as monotherapy or in combination with other therapeutic modalities. Allogeneic cancer vaccines offer several theoretical advantages, including ready availability, a broad and shared antigenic repertoire, and standardized, scalable manufacturing processes [16,21]. A particular strength of this approach is the ability to present multiple shared TAAs using reproducible production platforms derived from established tumor cell lines, enabling large-scale manufacturing and long-term storage [16].

However, a major limitation of allogeneic vaccines is their lack of patient-specific neoantigens [22]. Shared TAAs are more likely to be subject to central or peripheral immune tolerance and may therefore induce weaker, less tumor-specific T-cell responses [16]. Considering the clinical trial data discussed above, further research is needed to define the optimal disease stage, timing, and rational combination strategies required to enhance the therapeutic efficacy of allogeneic vaccine approaches.

#### 2.1.3. Dendritic Cell Vaccines

DCs are highly potent antigen-presenting cells that have been investigated as components of tumor vaccines since their initial identification by Steinman and colleagues in 1973 [23]. Steinman’s seminal work established DCs as a critical link between innate and adaptive immunity, by demonstrating their ability to sense danger-and pathogen-associated signals, migrate to lymphoid tissues, and prime naïve T cells via endogenous antigen processing and cross-presentation on MHC molecules [24]. By the late 1990s, these properties led to the hypothesis that DCs could be therapeutically exploited as cancer vaccines. Early clinical translation was limited by the scarcity of DCs, a barrier that was overcome with the discovery that bipotential CD34^+^ hematopoietic progenitor cells can differentiate into DCs [25]. This advance enabled large-scale DC production for clinical trials [26]. Subsequent studies have further revealed the substantial functional heterogeneity within DC populations.

DCs play essential roles in both innate and adaptive immune responses and are activated by exogenous danger signals, including pathogen-associated molecular patterns (PAMPs) and damage-associated molecular patterns (DAMPs) [27]. Following activation, DCs engage in cross-presentation, processing TAAs and presenting them on MHC class I molecules [28]. These antigen-loaded DCs subsequently cross-prime naïve T cells, generating antigen-specific effector T-cell responses after vaccine administration [27].

**Figure 3:**
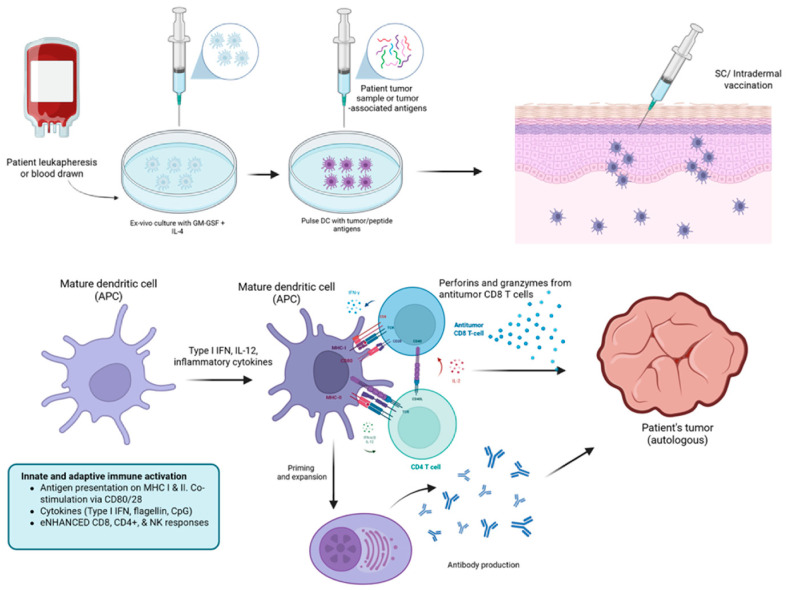
Mechanism of action for dendritic cell. Created in BioRender. Flores Banda, J. (2026). https://BioRender.com/dcab65k (accessed on 8 February 2026). GM-CSF, DC, SC, APC, IFN, IL-12, MHC: Major histocompatibility complex, CpG: Cytosine-phosphate-Guanine, NK: Natural killer cell.

Two principal strategies have been developed for DC-based vaccine generation: ex vivo and in vivo approaches. In the more commonly used ex vivo method, DCs are generated by differentiating CD14^+^ monocytes or CD34^+^ hematopoietic stem and progenitor cells collected by leukapheresis. Cells are cultured in the presence of GM-CSF and interleukin-4 to promote DC differentiation and maturation, while being simultaneously pulsed with TAAs. The resulting mature, antigen-loaded DCs are then administered back to the patient as a therapeutic vaccine [29], as outlined in Figure 3. In contrast, in vivo strategies aim to directly target endogenous DCs by coupling tumor antigens to monoclonal antibodies specific for DC surface receptors, thereby facilitating antigen uptake and presentation in situ [29].

Clinical evaluation of DC-based cancer vaccines has yielded mixed but informative results. The most notable randomized phase 3 trial was the IMPACT study, in which Kantoff et al. evaluated sipuleucel T, an autologous DC-based vaccine, in patients with castration-resistant prostate cancer [30]. Median OS was 25.8 months in the sipuleucel-T group compared with 21.7 months in the placebo group, despite no significant difference in time to disease progression. These findings led to U.S. Food and Drug Administration (FDA) approval of sipuleucel-T, establishing DC-based vaccination as a viable therapeutic modality [31].

In glioblastoma, Liau et al. reported results from a phase 3 prospective trial evaluating the addition of an autologous tumor lysate-loaded DC vaccine (DCVax-L) to standard therapy [32]. Median OS was 19.3 months in the experimental arm versus 16.5 months in controls (HR = 0.80; 98% CI, 0.00–0.94; *p* = 0.002). Among patients with recurrent disease, DCVax-L treatment was associated with a median OS of 13.2 months compared with 7.8 months for standard-of-care therapy.

Outcomes in melanoma have been more variable. A 2024 meta-analysis evaluating DC vaccines as adjuvant therapy following surgical resection demonstrated improved three-year OS compared with control treatments [33]. However, a phase 3 trial by Bol et al. assessing adjuvant DC therapy in stage IIIB/C melanoma reported inferior RFS in the vaccine arm compared with controls (median RFS 12.7 vs. 19.9 months) [34]. Overall, response rates to DC-based vaccines remain modest, though survival benefits have been observed in select settings. Future studies will need to evaluate the efficacy of DC vaccines in combination with modern therapeutic approaches such as immune checkpoint inhibitors and targeted therapies.

DC-based vaccines have consistently demonstrated an acceptable safety profile across clinical trials. Owing to their capacity to activate both CD4^+^ and CD8^+^ T lymphocyte responses, they elicit robust tumor antigen-specific immunity, which likely underlies the clinical benefit observed in select settings [35]. Nevertheless, several limitations persist, including complex manufacturing processes, immunosuppression within the TME, and reduced efficacy in advanced disease states [36,37]. Overcoming these barriers will be essential to improving the clinical impact of dendritic-cell--based immunotherapies. From a translational perspective, dendritic cell vaccines occupy a distinctive niche among therapeutic cancer vaccine platforms. Compared with peptide, DNA, ribonucleic acid (RNA), and viral vector vaccines, they offer highly efficient antigen presentation and can prime both CD4^+^ and CD8^+^ T-cell responses even in settings with poor baseline immunogenicity. However, these immunologic advantages are offset by major practical limitations, including leukapheresis requirements, individualized ex vivo manufacturing, batch-to-batch variability, high cost, and limited scalability across treatment centers. These factors help explain why, despite encouraging signals in selected tumors and the approval of sipuleucel-T, broader clinical adoption has remained limited relative to off-the-shelf vaccine platforms.

### 2.2. Peptide-Based Vaccines

In 1991, van der Bruggen et al. described antigens expressed by melanoma tumors that were recognized by cytolytic T lymphocytes identifying a potential for targeted immunotherapy against these antigen [38]. Then, in 1995, Marchand et al. reported tumor regression using this method in melanoma patients treated with a peptide encoded by gene MAGES-3 [39]. Following these early trials, numerous studies have since reported on the immunological efficacy of peptide vaccines to be discussed below.

**Figure 4:**
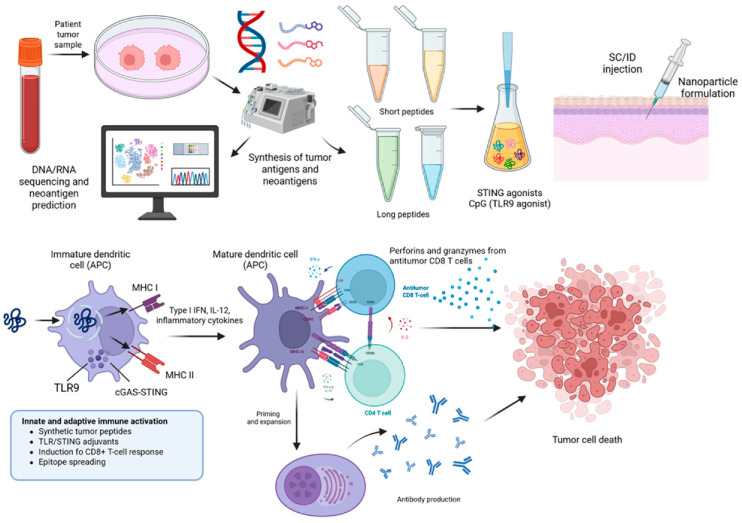
Mechanism of action for peptide vaccine. Created in BioRender. Flores Banda, J. (2026). https://BioRender.com/9k1z4br (accessed on 8 February 2026). SC, APC, IFN, IL-12, MHC, CpG, DNA: Deoxyribonucleic acid, RNA: Ribonucleic acid, TLR9: Toll-like receptor-9, ID: intradermal injection, cGAS: cyclic GMP-AMP synthase, STING: Stimulator of Interferon Genes.

Tumor antigens can be classified as TAAs or tumor-specific antigens (TSAs). TAAs are overexpressed in tumor cells but have low levels of expression in normal tissues, which can limit their immunogenicity. On the other hand, TSAs or neoantigens are highly immunogenic because they arise from tumor-specific mutations, can escape immune tolerance, and are recognized as non-self by immune cells [40,41]. They originate from genetic changes in cancer cells [42]. Peptide cancer vaccines are designed based on these neoantigens to elicit humoral and cellular immune responses targeting TSAs [43]. Figure 4 below describes the process of peptide vaccine production. The development of next-generation sequencing subsequently provided a means to identify immunogenic neoantigens in mouse tumor models in 2012 [44]. Neoantigens can further be classified into shared neoantigens and personalized neoantigens. Shared neoantigens refer to mutated antigens common across different cancer patients, while personalized neoantigens vary from patient to patient. Thus, the personalized neoantigen preparation can only be specifically targeted to an individual patient [45].

Personalized neoantigen vaccines represent a relatively recent therapeutic approach and remain in the early stages of clinical investigation. Weber et al. evaluated the combination of a personalized neoantigen vaccine with pembrolizumab and reported an 18-month RFS of 79% (95% CI, 69.0–85.6) for combination therapy compared with 62% (95% CI, 46.9–74.3) for pembrolizumab monotherapy, with comparable rates of immune-mediated adverse events, supporting the feasibility and potential added benefit of combining neoantigen vaccination with immune checkpoint inhibitors [46].

In a phase 1 study, Braun et al. investigated a personalized neoantigen-targeted cancer vaccine in patients with high-risk, fully resected clear cell RCC, administered alone or in combination with ipilimumab [47]. Following a median postoperative follow-up of 40.2 months, none of the nine treated patients experienced disease recurrence, and all mounted vaccine-specific T-cell immune responses, although the small cohort size limits definitive efficacy conclusions. The PNeoVCA phase 1 trial is currently ongoing to assess the safety of a personalized neoantigen peptide-based vaccine in combination with pembrolizumab for patients with advanced solid tumors [48]. Additionally, an active randomized phase 1/2 trial (NCT04183166) is evaluating a therapeutic cancer vaccine in patients with squamous cell carcinoma of the head and neck [49]. Among 33 patients randomized and followed for a median of 28.5 months, all 16 patients treated with the TG4050 vaccine remained disease-free, whereas 3 of 16 patients (19%) in the control arm experienced relapse [50]. The vaccine was well tolerated, with treatment-related adverse events primarily mild to moderate in severity.

Synthetic peptide-based vaccines offer several advantages over other personalized immunotherapy platforms, including relatively straightforward manufacturing and stability under standard storage conditions, which together contribute to favorable cost-effectiveness [51]. These vaccines can induce both CD8^+^ and CD4^+^ T-cell responses; however, rapid peptide degradation and the typically transient, low-magnitude immune responses have limited their clinical antitumor efficacy [51]. One ongoing area of investigation is the optimal design of peptide vaccines, particularly peptide length. Short peptides, typically 8–10 amino acids, can directly bind MHC class I molecules but may result in nonspecific antigen presentation and suboptimal T-cell priming [43]. In contrast, longer peptides (often >20 amino acids) require processing by professional APCs, leading to more effective T-cell activation and the induction of robust and durable antitumor immune responses [52]. Additional factors that limit the therapeutic efficacy of neoantigen vaccines include impaired antitumor immunity in patients with cancer-related immune dysfunction and tumor-intrinsic features such as low tumor mutational burden and pronounced intratumoral heterogeneity [52]. In comparison with cell-based and nucleic acid--based platforms, peptide vaccines offer several translational advantages, including relatively low manufacturing cost, chemical stability, ease of storage, and compatibility with off-the-shelf production. However, their clinical efficacy is often constrained by human leukocyte antigen (HLA) restriction, dependence on accurate epitope selection, and the tendency to induce weaker or less durable T-cell responses than viral vector or mRNA-based approaches. As a result, peptide vaccines may be particularly attractive in combination regimens, but as monotherapy they have generally shown more limited and less consistent activity than the most promising personalized RNA vaccine platforms.

### 2.3. Nucleic Acid Vaccines

#### 2.3.1. DNA

DNA vaccines are composed of plasmid DNA encoding a protective antigen, enabling host cells to synthesize the antigen internally and thereby trigger both humoral and cellular immune responses [53]. The concept arose in the 1960s, when naked DNA was first shown to transfect mammalian cells in vivo, and a landmark 1992 study demonstrated that such transfection could induce antigen-specific antibodies in animals [54,55,56]. These early breakthroughs led to the licensing of several veterinary DNA vaccines against infectious diseases and cancers [53]. In 2021, India authorized the SARS-CoV-2 vaccine ZyCoV-D for emergency use, representing the first DNA vaccine deployed in humans, although none has yet received FDA approval in the United States [57].

**Figure 5:**
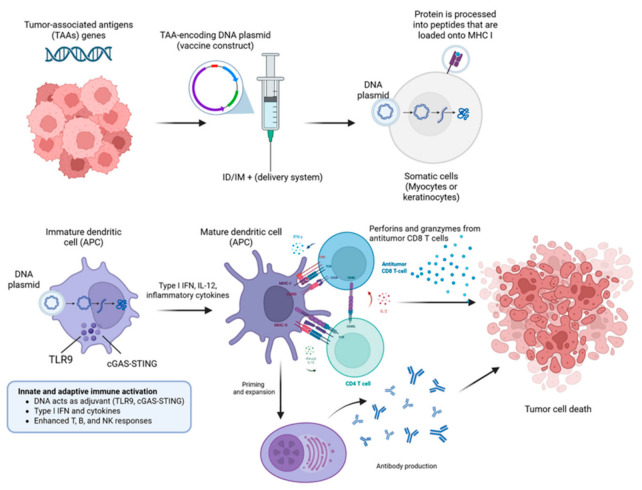
Mechanism of action for DNA vaccine. Created in BioRender. Flores Banda, J. (2026). https://BioRender.com/t78seot (accessed on 8 February 2026). IM: Intramuscular injection, APC, IFN, IL-12, MHC, DNA, TLR9, ID, cGAS, STING, NK, TAAs.

In oncology, DNA cancer vaccines are engineered to deliver plasmid DNA encoding TAAs—proteins that are preferentially or aberrantly expressed by malignant cells—thereby enabling in situ antigen production within the patient’s own cells [58]. This approach is intended to stimulate robust CD8^+^ cytotoxic T cell responses while also promoting humoral immunity against tumor-specific targets, as illustrated in Figure 5 [58]. Although DNA vaccines offer several practical advantages, including favorable safety profiles, molecular stability, and relatively low manufacturing costs, their clinical development has historically been limited by suboptimal immunogenicity in humans [54].

Preclinical studies have identified numerous mechanisms that contribute to resistance, including antigen loss or epitope modification, antigen-specific immune tolerance, T cell exhaustion, and the recruitment of immunosuppressive cell populations such as regulatory T cells (Tregs), myeloid-derived suppressor cells, and tumor-associated macrophages. Additional barriers include the production of inhibitory cytokines (e.g., transforming growth factor beta and Interleukin-10), metabolic constraints within the TME, clonal selection of immune escape variants, and the intrinsically immunosuppressive biology of many tumors [59].

To address these limitations, contemporary strategies have focused on optimizing both antigen design and vaccine delivery. Physical delivery techniques such as electroporation (EP) transiently increase cell membrane permeability, enhancing plasmid uptake by approximately 100 to 1000 fold compared with naked DNA injection and substantially increasing antigen expression [60,61]. Clinically, EP-assisted delivery has been associated with improved immunogenicity. For instance, an EP-enabled prostate specific membrane antigen vaccine induced interferon γ-producing CD8^+^ T cell responses in approximately 60% of treated patients and produced more than a 14-fold increase in antibody titers compared with DNA administration without EP. Similarly, an EP-delivered human papillomavirus (HPV) 16/18 therapeutic DNA vaccine elicited strong humoral and cellular immune responses that exceeded those observed with earlier HPV DNA, peptide, and poxviral vaccine platforms [60].

Alternative physical delivery approaches, including intradermal gene gun administration, have also demonstrated enhanced immunogenicity. In a HER2/neu murine tumor model, gene gun--mediated vaccination resulted in long-term, tumor-free survival in approximately 60% of animals, compared with roughly 25% survival when the same plasmid was delivered using a jet injector [62].

Another promising avenue involves combining DNA vaccines with immune checkpoint inhibitors. In prostate cancer, early trials of the pTVG-HP DNA vaccine targeting prostatic acid phosphatase induced PAP specific CD4^+^ and CD8^+^ T cell responses, yet vaccination alone failed to improve two-year metastasis free survival compared with control (41.8% vs. 42.3%, *p* = 0.97) [63,64]. In contrast, combination therapy with pTVG HP and the programmed cell death protein 1 (PD-1) inhibitor nivolumab significantly prolonged prostate-specific antigen doubling time, underscoring the synergistic potential of DNA vaccination when paired with immune checkpoint blockade [65].

Collectively, these findings suggest that DNA cancer vaccines can function as effective systemic therapies, including in metastatic disease, when integrated with rational antigen selection, optimized physical delivery platforms, and complementary immunotherapeutic agents. Compared with RNA vaccines, DNA platforms offer greater molecular stability, lower manufacturing cost, and less stringent storage requirements, which favor broad distribution and repeated dosing. However, in contrast to both RNA and viral vector--based vaccines, DNA vaccines have historically shown weaker immunogenicity in humans and often require device-assisted delivery methods such as EP to achieve clinically meaningful antigen expression. Thus, the major translational strength of DNA vaccines lies in manufacturability and safety, whereas their principal limitation remains the need to consistently generate potent antitumor T-cell responses in patients.

#### 2.3.2. RNA

RNA vaccines function by delivering synthetic mRNA into the cytoplasm, where host ribosomes translate it into disease-specific proteins that stimulate both cellular and humoral immune responses [66]. Early development was limited by RNA instability, rapid enzymatic degradation, and excessive innate immune activation, but these barriers were substantially overcome by improved mRNA engineering and delivery systems, particularly lipid nanoparticles [67]. The clinical success of mRNA vaccines during the COVID-19 era subsequently validated this platform and accelerated its application in cancer immunotherapy [68].

**Figure 6:**
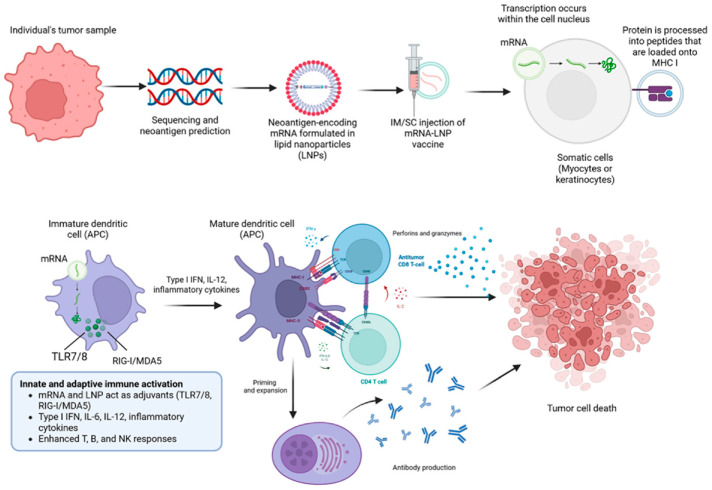
Mechanism of action for RNA vaccine production. Created in BioRender. Flores Banda, J. (2026). https://BioRender.com/clsc999 (accessed on 8 February 2026). IM, APC, IFN, IL-12, IL-6: Interleukin-6, MHC, mRNA: Messenger ribonucleic acid, TLR7/8: Toll-like receptor-7 and toll-like receptor-8, SC, LNPs: Lipid nanoparticles, NK, RIG-I: Retinoic acid-Inducible Gene-I, MDA5: Melanoma Differentiation-Associated protein 5.

The same technological advances underpinning infectious disease mRNA vaccines have been adapted for cancer immunotherapy, as illustrated in Figure 6, where preclinical studies show that LNP-formulated mRNA constructs can induce potent CD8^+^ T-cell responses, enhance tumor control, and synergize with immune checkpoint blockade [69,70]. These findings support the biologic rationale for applying RNA vaccine platforms in both shared-antigen and personalized neoantigen settings.

Notably, spike mRNA LNP formulations analogous to COVID-19 vaccines can independently convert immunologically “cold” tumors into lesions responsive to PD-1/programmed death ligand 1 (PD-L1) inhibition through a type I interferon-dependent mechanism that enhances antigen presentation and tumor-reactive CD8^+^ T cell infiltration. In non-small cell lung cancer cohorts, COVID-19 vaccination within 100 days of initiating immunotherapy was associated with prolonged OS (33.3 months vs. 20.6 months) [71].

Early clinical application of mRNA cancer vaccines in melanoma involved direct intradermal injection of autologous tumor-derived mRNA, a strategy that proved feasible and safe in a first in human trial [72]. Subsequently, the randomized phase 2b KEYNOTE 942 study demonstrated that the personalized neoantigen vaccine mRNA 4157 (V940) combined with pembrolizumab significantly improved RFS (HR 0.56; 95% CI, 0.309–1.017; two-sided *p* = 0.053), with an 18 month RFS of 79% (95% CI, 69.0–85.6) compared with 62% (95% CI, 46.9–74.3) for pembrolizumab alone and a manageable safety profile [46].

Building on these findings, the RNA LPX vaccine FixVac (BNT111), which encodes four shared TAAs (NY ESO 1, MAGE A3, tyrosinase, and TPTE), has demonstrated robust immunogenicity in advanced melanoma. More than 75% of evaluable patients develop detectable CD4^+^ and CD8^+^ T cell responses, with de novo cytotoxic CD8^+^ clones expanding to high frequencies and acquiring PD-1^+^ effector memory phenotypes capable of tumor cell lysis [73]. In combination with anti PD-1 therapy, BNT111 has achieved objective tumor regression in over one third of patients previously treated with checkpoint inhibitors, with response rates approaching 50% at the 100 µg dose level [73].

Beyond antigen-specific vaccination, multi-target immunomodulatory mRNA LNP platforms are being developed to reprogram the immunosuppressive TME. In a pilot clinical study involving dogs with spontaneous malignancies, an mRNA LNP vaccine targeting seven immunosuppressive mediators (CCL22, TGF-β, CTLA-4, galectin 3, PD-L1, IDO1, and ARG1) was well tolerated and resulted in stable disease in 75% of treated animals, along with durable progression-free survival and improvements in paraneoplastic metabolic and hematologic abnormalities, highlighting the translational promise of TME-directed RNA vaccines [74].

RNA cancer vaccines offer several practical and biological advantages. Once tumor antigens or neoantigens are identified, vaccine design and in vitro transcription can be rapidly performed at scale, without reliance on cell culture systems or viral vectors [66]. In a phase 1 adjuvant trial, the individualized mRNA vaccine autogene cevumeran (BNT122/RO7198457) was manufactured from resected pancreatic ductal adenocarcinoma tissue and administered within approximately nine weeks of surgery, successfully meeting predefined workflow timelines [75]. Building on these findings, updated long-term results from this trial were presented at the 2026 American Association for Cancer Research Annual Meeting [76]. The vaccine, encoding up to approximately 20 patient-specific neoantigens was administered following surgical resection with chemotherapy and the PD-1 inhibitor atezolizumab, and induced robust neoantigen-specific CD8^+^ T-cell responses in 50% (8/16) of treated patients. Among immune responders, 87.5% (7/8) were alive at approximately six years of follow-up and the median OS had not yet been reached, whereas non-responders demonstrated a median OS of roughly 3.4 years, suggesting that vaccine-induced T-cell immunity may translate into a durable survival benefit in selected patients. These data challenge the longstanding view of pancreatic ductal adenocarcinoma as a non-immunogenic tumor, although the small cohort size, the need for complex sequencing-driven manufacturing, and the restriction of benefit to immune responders underscore the need for larger, controlled studies before this approach can be considered practice-changing [76].

A subsequent phase 1 study evaluating autogene cevumeran alone and in combination with atezolizumab in advanced solid tumors demonstrated favorable tolerability and neoantigen-specific CD4^+^ and CD8^+^ T-cell responses in 71% of patients, frequently arising de novo and persisting for up to 23 months. Vaccine-induced CD8^+^ T cells constituted a median of 7.3% of circulating T cells and up to 7.2% of tumor infiltrating lymphocytes, with early indications of clinical activity and a best case manufacturing turnaround of 28 days [77]. The Mutanome Engineered RNA Immunotherapy platform exemplifies next-generation strategies that integrate premanufactured libraries of shared TAAs with patient-specific neoantigen mRNAs, enabling treatment of aggressive malignancies such as triple negative breast cancer [78]. Compared with DNA vaccines, RNA platforms do not require nuclear entry and generally permit faster design-to-manufacturing timelines, making them particularly attractive for personalized neoantigen vaccine development. In contrast to viral vector--based vaccines, mRNA formulations also avoid anti-vector immunity and are more readily re-dosed, while preserving the ability to induce robust T-cell responses. However, these advantages are counterbalanced by major translational challenges. Fully personalized RNA vaccines depend on high-quality tumor sampling, rapid next-generation sequencing, accurate neoantigen prediction, and tightly coordinated manufacturing workflows, all of which increase cost and limit scalability. In addition, vaccine efficacy may be undermined by false-positive neoantigen prediction, HLA-binding variability, low neoantigen clonality, antigen loss under immune pressure, and marked intratumoral heterogeneity. These factors likely contribute to the discordance often observed between vaccine immunogenicity and durable clinical benefit and underscore the need for improved computational prioritization, biomarker-driven patient selection, and combination strategies.

Because mRNA vaccines remain episomal and are rapidly degraded following protein translation, they eliminate the risk of genomic integration and generally exhibit favorable safety profiles. Nonetheless, innate immune sensing of both mRNA and LNP components can either augment or hinder antitumor responses, underscoring the importance of carefully optimizing mRNA chemistry and delivery systems to maximize therapeutic efficacy while minimizing excessive inflammation.

### 2.4. Viral Vector-Based Vaccines

Viral vector-based vaccines represent an emerging class of cancer immunotherapies that utilize genetically engineered viruses to deliver tumor antigens, thereby inducing robust immune activation and promoting tumor-specific T-cell responses [79]. Early development began with recombinant poxvirus platforms in the 1990s and later expanded to adenoviral vectors. These approaches harness the intrinsic immunogenicity of viral infection to enhance antigen presentation and CD8^+^ T-cell priming, although clinical efficacy as monotherapy has been variable [9].

To ensure safety, viral genomes are genetically modified to remove or inactivate genes essential for viral replication and pathogenicity. As a result, these vectors are nonpathogenic and incapable of generating new infectious particles in patients, while still retaining the ability to infect host cells and elicit strong immune responses [80]. This design enables efficient tumor antigen expression without causing clinical infection.

**Figure 7:**
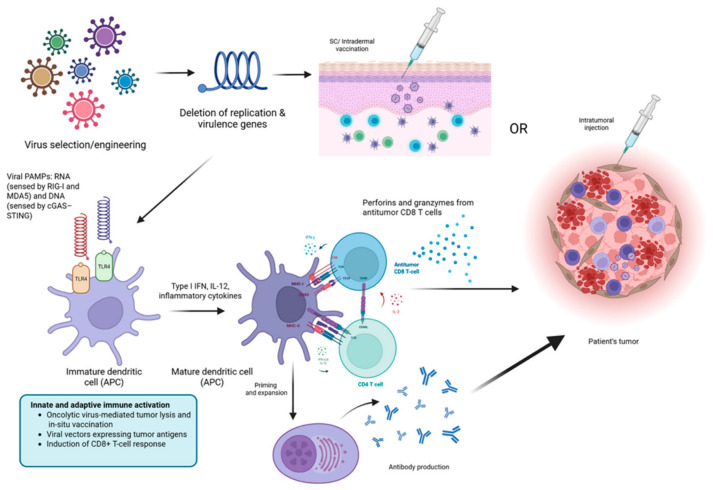
Mechanism of action for viral based vaccine. Created in BioRender. Flores Banda, J. (2026). https://BioRender.com/s0hr8dx (accessed on 8 February 2026). APC, IFN, IL-12, RNA, SC, RIG-I, MDA5, cGAS, STING, PAMPs: Pathogen-Associated Molecular Patterns.

Viral vector vaccines stimulate antitumor immunity through complementary innate immune mechanisms. First, infection of host cells induces a localized inflammatory response characterized by the release of pro-inflammatory cytokines and chemokines, which recruit innate immune cells and promote tumor-antigen-specific T cell priming [81]. This inflammatory milieu can partially counteract the immunosuppressive TME. Second, viral vectors provide PAMPs that are recognized by pattern recognition receptors, including toll-like receptors, triggering type I interferon-dependent signaling pathways. This cascade leads to dendritic cell maturation and enhanced antigen presentation, resulting in potent immune priming, as illustrated in Figure 7 [82,83].

For tumor control to occur, this innate immune activation must ultimately drive effective adaptive immunity. Viral vector vaccines achieve this by promoting efficient antigen presentation: following infection, virally encoded tumor antigens are captured by DCs and presented on MHC class I molecules, activating tumor-specific CD8^+^ cytotoxic T cells [84]. Concurrently, antigen presentation on MHC class II molecules activates CD4^+^ helper T cells, which support cytotoxic function, memory formation, and sustained antitumor immunity [85]. This coordinated activation of innate and adaptive immune responses can also promote epitope spreading, broadening immune recognition beyond the initially targeted antigen [86]. Collectively, these mechanisms highlight the capacity of viral vector--based vaccines to generate multifaceted antitumor immune responses capable of at least partially overcoming an immunosuppressive TME, although important advantages and limitations remain to be addressed.

Viral vector--based cancer vaccines have been evaluated in multiple late phase clinical trials. In metastatic castration-resistant prostate cancer, the recombinant poxviral vaccine PROSTVAC demonstrated improved median OS in a randomized phase 2 trial (25.1 vs. 16.6 months; HR 0.56; *p* = 0.006) [87]. However, this survival benefit was not reproduced in the phase 3 PROSPECT trial, which showed no OS advantage compared with control (34.8 vs. 34.7 months; HR 1.01; *p* = 0.90) [88]. In contrast, durable clinical activity has been observed in melanoma. Andtbacka et al. reported results from a phase 3 trial comparing talimogene laherparepvec (T-VEC) with GM-CSF in unresectable melanoma, demonstrating a median OS of 23.3 months (95% CI, 19.5–29.6) in the T-VEC arm versus 18.9 months (95% CI, 16.0–23.7) in the control group, with complete responses observed in 16.9% of treated patients [89]. Based on durable responses and survival benefit, T-VEC received FDA approval for advanced melanoma.

More recently, adenoviral vector--based gene therapy has shown efficacy in urothelial carcinoma. Boorjian et al. reported results of a phase 3 trial evaluating nadofaragene firadenovec vncg, a nonreplicating adenoviral vector delivering the human interferon alfa-2b gene, thereby driving local cytokine production in patients with BCG-unresponsive non-muscle invasive bladder cancer [90]. Among patients with carcinoma in situ, 53.4% achieved a complete response within three months, and 45.5% maintained the response at 12 months. These findings led to FDA approval in 2022 as the first intravesical gene therapy for this population [91]. Five-year follow-up data demonstrated an OS rate of 80% (95% CI, 71.0–86.0) and cystectomy-free survival of 49% (95% CI, 40.0–57.1), supporting durable clinical benefit [92]. Together, these data underscore the ability of viral vectors to induce clinically meaningful immune responses, while also highlighting the inconsistency of long term survival benefit when used as standalone therapies.

Viral vector--based vaccines offer several advantages, including efficient in vivo antigen delivery without pathogenicity, potent innate and adaptive immune activation, and genetic flexibility that allows rapid insertion or modification of tumor antigens, facilitating personalized vaccine design and scalable manufacturing [81]. However, notable limitations exist. Pre-existing or treatment-induced anti-vector immunity may reduce transgene expression and impair efficacy with repeat dosing [81,93]. Additionally, when administered as monotherapy, viral vaccines have generally shown modest activity in solid tumors, likely due to tumor mediated immunosuppression [81]. Collectively, these observations support the rationale for combination strategies—particularly with immune checkpoint inhibitors—to enhance the therapeutic potential of viral vector--based cancer vaccines [94]. Compared with DNA and RNA platforms, viral vector--based vaccines benefit from highly efficient in vivo antigen delivery and strong intrinsic adjuvanticity, which can enhance dendritic cell activation and T-cell priming. This may provide an advantage in tumors requiring stronger innate immune stimulation, and it likely contributes to the durable clinical activity observed with selected agents such as T-VEC and nadofaragene firadenovec. However, these platforms are also constrained by anti-vector immunity, more complex manufacturing and quality-control requirements, and less flexibility for rapid patient-specific customization than personalized RNA vaccines. Accordingly, viral vector vaccines currently appear most advantageous when potent in situ immune activation is desired, whereas RNA platforms may be better suited to individualized neoantigen targeting.

### 2.5. Bacterial-Based Vaccines

Despite advances in existing vaccine platforms, challenges related to efficient tumor targeting and adequate antigen delivery persist, driving ongoing efforts to develop more effective vaccine delivery systems. Live bacterial vectors have emerged as a promising area of research in cancer immunotherapy [95]. The concept of harnessing infection-induced immune activation to treat cancer dates back over a century. As described by Bickels et al., Dr. William B. Coley pioneered this approach in the early 1900s by administering injections containing Serratia marcescens and Streptococcus species to patients with soft tissue sarcomas, lymphomas, osteosarcomas, and melanomas, observing tumor regression in a subset of cases [96].

Modern bacterial-based cancer vaccines build upon this historical foundation by leveraging live attenuated bacteria, inactivated bacteria, bacterial components, or genetically engineered bacterial vectors to stimulate antitumor immune responses. These platforms are particularly attractive due to the intrinsic ability of certain bacteria to preferentially colonize tumors and proliferate within hypoxic and necrotic regions of the TME [95].

Similar to other cancer vaccine strategies, live bacterial vaccines mediate tumor elimination through activation of APCs and subsequent induction of adaptive immune responses. Engineered bacteria are designed to carry TAAs, which are recognized and processed by APCs. Following administration, the bacteria preferentially localize to and colonize tumor tissue, where they proliferate and express therapeutic payloads. Antigen processing and presentation then activates CD4^+^ and CD8^+^ T cells, triggering cytotoxic immune responses and tumor cell apoptosis once tumor antigens are recognized [95].

The most well-established example of bacterial immunotherapy is BCG, which remains the standard of care for non-muscle invasive bladder cancer [97]. Antonelli et al. demonstrated that BCG therapy enhances the effector function of tumor-specific CD4^+^ T cells through interferon γ production [98]. A systematic review by Shelley et al., encompassing six trials and 585 patients, reported a pooled HR for first recurrence of −0.83 (95% CI, −0.57–1.08), corresponding to a 56% reduction in recurrence risk when BCG was combined with transurethral resection compared with surgery alone at 12 months [99]. Building on the success of BCG, additional early-stage clinical trials evaluating live bacterial therapies are currently underway for multiple myeloma, pancreatic cancer, small cell lung cancer, bladder cancer, and cervical cancer [95].

Among bacterial platforms, Salmonella species have demonstrated superior immunogenicity compared with inactivated vaccines, making them attractive candidates for tumor vaccination [100]. However, given the pathogenic nature of Salmonella, vaccine development has focused on engineering attenuated strains to ensure safety. Wu et al. described a novel live bacterial vaccine platform in which attenuated Salmonella was coated with a cancer cell membrane, thereby combining potent bacterial adjuvanticity with TSAs; this strategy induced durable immune responses and tumor suppression across multiple murine models [101]. Earlier work by Zhao et al. demonstrated the antitumor efficacy of the Salmonella typhimurium A1 R mutant in mouse models of metastatic human prostate cancer [102]. Additional bacterial vectors under investigation include Listeria monocytogenes, Clostridium novyi, and Escherichia coli [103,104,105]. Achieving effective molecular delivery while maintaining an acceptable safety profile remains essential to the successful translation of bacterial vaccine platforms.

**Figure 8:**
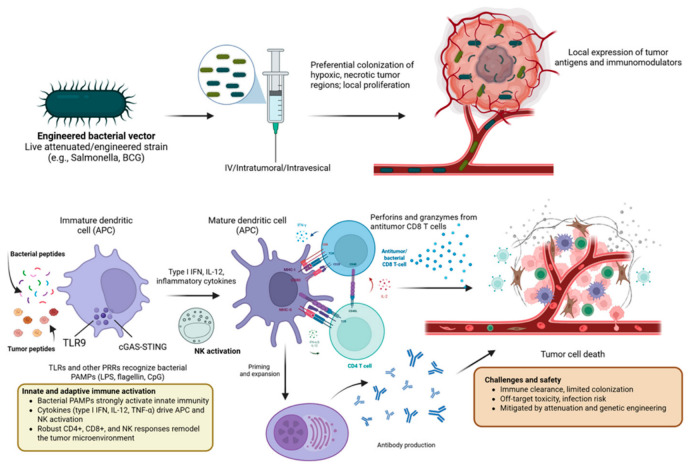
Mechanism of action for bacterial based vaccine. Created in BioRender. Flores Banda, J. (2026). https://BioRender.com/h5jzw14 (accessed on 8 February 2026). IV: Intravenous, BCG: Bacille Calmette-Guerin, APC, IFN, IL-12, TLR9, cGAS, STING, PAMPs, TLRs: Toll-like receptors, NK, TNF: Tumor Necrosis Factor.

Bacterial vectors offer several intrinsic advantages, including ease of genetic manipulation, the capacity for in vivo proliferation, and preferential tumor targeting. As illustrated in Figure 8, tumor tropism represents the principal advantage of bacterial-based vaccines. Anaerobic bacteria such as Salmonella typhimurium naturally localize to hypoxic, nutrient-rich TMEs, achieving significantly higher tumor-to-normal tissue ratios and enabling deeper penetration into solid tumors, where conventional therapies may be ineffective [95]. Nevertheless, several challenges remain, including rapid immune clearance, incomplete or heterogeneous tumor colonization, off-target toxicities, and safety concerns [95]. Among these, safety remains the most significant limitation, given the inherent risk of infection associated with live bacterial administration [106]. Ongoing research is therefore focused on refining bacterial engineering strategies to enhance safety while preserving or augmenting therapeutic efficacy. Relative to other vaccine platforms, bacterial vectors offer the distinctive advantage of preferential tumor colonization, particularly within hypoxic and necrotic regions that are difficult for many systemic therapies to penetrate. This feature may enable localized immune activation and payload delivery in TMEs that are poorly accessible to peptide or nucleic acid vaccines. At the same time, the translational path for bacterial platforms is complicated by biosafety concerns, infection risk, regulatory complexity, and challenges in reproducible manufacturing, which currently make them less mature than viral vector and RNA-based approaches for broader oncology applications.

## 3. Discussion

As discussed throughout this review, cancer vaccine strategies have evolved markedly over the past several decades, transitioning from empiric whole cell approaches to increasingly rational and personalized platforms. Current vaccine modalities-including peptide-based, nucleic acid--based, dendritic cell, whole-cell, and viral or bacterial vector--based vaccines-reflect significant advances in our understanding of tumor immunology, antigen processing, and immune activation. Taken together, clinical experience with these diverse platforms highlights both the breadth of available strategies and the persistent gaps in achieving durable, widely applicable antitumor efficacy.

Despite advances in vaccine design and the ability of cancer vaccines to induce robust immune responses, clinical efficacy has remained limited in many studies. Multiple, overlapping mechanisms of immune resistance within the TME account for this inconsistency. Regulatory T cells and myeloid-derived suppressor cells, which accumulate in the TME, suppress effector T-cell function and promote resistance to vaccine-induced and other forms of immunotherapy [107]. Myeloid-derived suppressor cells inhibit antitumor immunity through the production of cytokines and modulation of immune-cell interactions, whereas regulatory T cells suppress cytotoxic immune responses and facilitate tumor growth [108]. Cancer-associated fibroblasts represent another major barrier to vaccine efficacy by establishing a physical and biochemical barrier that restricts T cell infiltration into tumors [109]. Metabolic alterations within the TME further impair vaccine-mediated antitumor immunity. Competition for nutrients and the accumulation of metabolic byproducts impair T cell activation and effector functions, while simultaneously promoting the expansion of immunosuppressive cell populations [110]. In addition, antigen heterogeneity and immunoediting substantially limit vaccine responses. During immunoediting, highly immunogenic subclones are eliminated by antitumor T-cells responses, leading to the emergence of less immunogenic populations. Tumors that evade complete immune elimination often develop intratumoral heterogeneity, resulting in the coexistence of multiple genetically distinct cancer cell populations. These tumors are associated with poor patient outcomes and lack response to immunotherapies [111]. The effectiveness of personalized vaccines is further compromised by prolonged manufacturing timelines. Tumor evolution and ongoing immunoediting between biopsy and vaccine administration may alter neoantigens, potentially rendering selected targets less effective [112]. Importantly, these resistance mechanisms do not function independently but instead interact to reinforce the immunosuppressive nature of the TME. Consequently, overcoming these barriers will likely require combination strategies that simultaneously target multiple pathways of immune resistance. In support of this approach, Imani et al. proposed that targeting metabolic and immunological pathways through combination strategies, including checkpoint blockade, chimeric antigen receptor T-cell therapies, and microbiome modulation, may transform the breast TME from an immunologically “cold” state to a more immunogenic and responsive microenvironment [113].

Looking ahead, the integration of cancer vaccines with standard treatment modalities—particularly immune checkpoint inhibitors—will be critical to enhancing their clinical impact. Accumulating evidence suggests that, in most solid tumors, vaccines used as monotherapy are unlikely to achieve durable responses for the majority of patients. In contrast, combination strategies incorporating immune checkpoint blockade, targeted therapies, chemotherapy, or radiation have shown greater promise in early-phase studies.

Cytotoxic T-lymphocyte-associated antigen 4 and PD-1 are key negative regulators of T-cell function that contribute to immune tolerance and tumor escape [114]. Cancer vaccines enhance antigen-specific immune activation and promote immune-cell infiltration into the TME, whereas immune checkpoint inhibitors restore and sustain T-cell effector function by preventing immune exhaustion and dysfunction. Together, these complementary mechanisms create a strong rationale for synergistic antitumor activity through enhanced immunogenicity and reduced immunosuppression [115,116]. Furthermore, as discussed previously, combination strategies involving vaccines and immune checkpoint inhibitors have demonstrated encouraging clinical activity across multiple tumor types, supporting their continued investigation.

Chemotherapy may also augment vaccine efficacy through the induction of immunogenic cell death of tumor cells, promoting the release of TAAs, TSAs and DAMPs. This process broadens the antigenic repertoire available for antigen presentation and T-cell priming while promoting a more favorable immune microenvironment [117]. Several commonly used chemotherapies, including anthracyclines, paclitaxel, and oxaliplatin, have been validated as potent immunogenic cell-death inducers and represent attractive partners for vaccine-based immunotherapy [118].

Similarly, radiotherapy can enhance antitumor immune responses by promoting antigen release, increasing cytokine and chemokine production, and facilitating dendritic-cell activation and T-cell priming [119]. In a poorly immunogenic murine model of triple-negative breast cancer, Lhuillier et al. demonstrated that vaccination with neoepitopes encoded by genes upregulated following radiotherapy improved tumor control, highlighting the potential for radiotherapy to generate novel immunogenic targets [120].

Oncolytic viruses represent another promising combination strategy. These agents selectively infect and lyse tumor cells, leading to the release of antigens and danger signals that stimulate both innate and adaptive immune responses [121]. Consistent with this concept, Koske et al. demonstrated that combining oncolytic virotherapy with cancer vaccination significantly enhanced antitumor efficacy, which was associated with increased T-cell activation and reduced regulatory T-cell infiltration within the TME [122]. As the understanding of tumor immunology continues to expand, rationally designed combination strategies are likely to become essential for overcoming the immunosuppressive barriers that currently limit vaccine efficacy.

Artificial intelligence--assisted approaches to antigen discovery, vaccine design, and patient selection have emerged as promising strategies to accelerate the development of effective cancer vaccines. One of the principal challenges in personalized cancer vaccine development is identifying which neoantigens can elicit a clinically meaningful immune response. Traditional neoantigen validation methods are resource-intensive, time-consuming and often inefficient. Consequently, artificial intelligence and machine learning technologies have gained considerable attention as tools for improving neoantigen prediction and streamlining vaccine development [123].

In spite of significant advances, accurate prediction of immunogenic neoantigens remains the major challenge. Current artificial intelligence-assisted prediction models are highly dependent on the quality and diversity of training datasets, and further optimization is needed to improve performance across tumor types and patient populations [124]. Promising results from American Society of Clinical Oncology 2025 by Cho et al. demonstrated 15.6% increase in the area under the precision-recall curve compared to existing methods for their machine learning model [125].

Beyond neoantigen identification, artificial intelligence is being applied to optimize mRNA vaccine design. Machine-learning approaches incorporating codon refinement and untranslated-region engineering have been shown to improve protein expression, stability and translation efficiency [126]. Furthermore, given the heterogeneity of immunotherapy responses, artificial intelligence has emerged as a valuable tool for immune subtyping and patient stratification, enabling identification of patients most likely to benefit from neoantigen vaccines [127].

Recent studies highlight the potential of these approaches. For example, Vasudevan et al. categorized patients with colorectal cancer by immune-gene activity using a machine-learning-driven framework to identify those who could potentially benefit most from neoantigen-based vaccines [128]. Such advances demonstrate that artificial intelligence may play a critical role not only in vaccine design but also patient optimization and treatment personalization.

Despite the rapid progress in artificial intelligence-assisted vaccine development, several major challenges remain. Model generalizability across diverse patient populations has been limited, and tumor heterogeneity may promote immune evasion thereby constraining prediction accuracy. Additional barriers include data scarcity and associated training biases, logistical constraints related to manufacturing timelines, and ongoing ethical and regulatory concerns regarding data privacy and governance [129]. As highlighted in recent reviews of next-generation neoantigen mRNA vaccines, persistent challenges remain related to HLA diversity, tumor heterogeneity, immunoediting, and manufacturing scalability for personalized vaccine platforms [130]. Addressing these limitations will be essential for the successful integration of artificial intelligence technologies into personalized cancer vaccine development and clinical practice. Continued investigation into rational combinations and advanced platform technologies will be essential to fully realize the therapeutic potential of cancer vaccination in clinical practice.

## 4. Conclusions

Therapeutic cancer vaccines have evolved into a variety of platforms-including cell-based, peptide-based, nucleic acid, and viral or bacterial vector approaches. Clinical trials show they are safe and reliably trigger tumor-specific immune responses, validating vaccination as an anticancer strategy. However, their effectiveness as monotherapy remains inconsistent, especially in advanced, immune-resistant cancers.

No single vaccine platform is superior; each has unique strengths and limitations that often complement each other. Cell-based vaccines offer personalized antigen presentation but face manufacturing and scalability challenges. Peptide and nucleic acid vaccines are easier and cheaper to produce but may be less effective due to immune constraints. Viral and bacterial vectors strongly activate immune responses but are limited by preexisting immunity, safety concerns, and inconsistent clinical outcomes.

These findings suggest vaccines should be used as precision immune primers in combination treatments, rather than as stand-alone agents. Vaccines can broaden antigen-specific T-cell responses, boosting the effectiveness of therapies like immune checkpoint inhibitors, chemotherapy, and radiotherapy. The success of personalized mRNA and neoantigen vaccines highlights the promise of tailoring strategies with advanced sequencing, immunology, and artificial intelligence-based antigen prediction.

To fully realize the potential of cancer vaccines, advances are needed in understanding resistance mechanisms, improving antigen selection and delivery, developing predictive biomarkers, and addressing manufacturing and equity challenges. As these barriers are overcome, cancer vaccines could become a central part of personalized, durable, and potentially curative cancer treatment.

## Data Availability

No new data were created or analyzed in this study. Data sharing is not applicable to this article.

## Abbreviations

The following abbreviations are used in this manuscript:

APCs	Antigen-Presenting Cells
BCG	Bacillus Calmette-Guerin
CI	Confidence Interval
DAMPs	Damage-associated molecular patterns
DCs	Dendritic Cells
DCVax-L	Dendritic Cell Vaccine-Lysate
DNA	Deoxyribonucleic acid
EP	Electroporation
FDA	U.S. Food and Drug Administration
GM-CSF	Granulocyte-macrophage colony-stimulating factor
HLA	Human Leukocyte Antigen
HR	Hazard Ratio
HSPPC 96	Heat Shock Protein-Peptide Complex 96
HPV	Human papillomavirus
MHC	Major Histocompatibility Complex
mRNA	Messenger RNA
OS	Overall Survival
PAMPs	Pathogen-Associated Molecular Patterns
PD-1	Programmed cell death protein 1
PD-L1	Programmed death ligand 1
RCC	Renal Cell Carcinoma
RFS	Recurrence-Free Survival
RNA	Ribonucleic Acid
TAAs	Tumor-Associated Antigens
TME	Tumor Microenvironment
TSAs	Tumor-Specific Antigens
T-VEC	Talimogene laherparepvec
